# Amputation risk from *Providencia stuartii* infection in a patient with diabetes mellitus: A case report

**DOI:** 10.1016/j.idcr.2025.e02391

**Published:** 2025-10-06

**Authors:** Yen-Ning Chang, Jian-Jr Lee, Chi Lo

**Affiliations:** a Department of Education, China Medical University Hospital, No. 2, Yude Road, North District, Taichung City 404327, Taiwan; b Department of Plastic and Reconstructive Surgery, China Medical University Hospital, No. 2, Yude Road, North District, Taichung City 404327, Taiwan

**Keywords:** *Providencia*, Diabetes mellitus, Wound infection, Bacteremia

## Abstract

We describe the emergence of *Providencia stuartii* infection and subsequent bacteremia in a bedridden patient with poorly controlled diabetes mellitus. Despite empirical treatment, the disease progressed, leading to septic shock and below-knee amputation. We examine the timing and clinical progression of the infection, highlighting that *P. stuartii* wound infections in diabetic patients can lead to severe complications. Early detection and appropriate intervention are crucial to prevent such outcomes.

## Highlight

• Diabetic wound infection progressing to bacteremia and amputation caused by *Providencia stuartii*

## Introduction

The genus *Providencia* is a urease-producing, gram-negative bacillus within the family Enterobacteriaceae. This genus includes five species: *Providencia stuartii*, *Providencia rettgeri*, *Providencia alcalifaciens*, *Providencia heimbachae*, and *Providencia rustigianii* [1], [2]. *Providencia* is an opportunistic pathogen which often causes infection in immunocompromised patients, such as diabetic patients, elders, infants, and long-term bedridden patients [3]. Human isolates of *Providencia* species have been recovered from urine, throat, perineum, axilla, stool, blood, and wound specimens [2]. Although the most common site of infection is the urinary tract, *Providencia stuartii* still sometimes causes severe wound infections and bacteremia [4]. *P. stuartii* appears to be sensitive to the antimicrobial agent imipenem. Fluoroquinolones, aminoglycosides, and fourth-generation cephalosporins are also likely to provide useful options for antibiotic therapy [5]. However, drug resistance is increasing recently, so the treatment should be based on the antimicrobial sensitivities, site of infection, cost and comorbid conditions [4], [5]. Due to the rarity of *P. stuartii* bacteremia developed from wound infections, few cases have been documented. This report presents a patient with poorly controlled type 2 diabetes mellitus (DM) who developed bacteremia from a wound infection of *P. stuartii*, highlighting the challenges in controlling the progression of sepsis.

## Case presentation

**Fig. 1:**
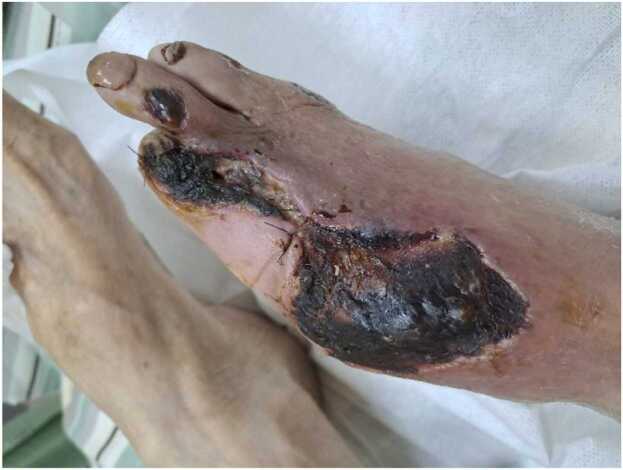
Despite amputation of the right first to third toes and repeated surgical debridement, the wound demonstrated delayed healing.

An 87-year-old bedridden patient presented with gangrene and bone necrosis of the right big toe was admitted through the emergency department. His medical history included stroke, prostate cancer, hypertension, and poorly controlled type 2 DM. During hospitalization, the patient underwent sequestrectomy and amputation of the right first to third toes. The wound was dressed daily but healed poorly (Fig. 1), requiring multiple bedside debridement. Amoxicillin/ Clavulanate 600 mg three times a day was prescribed initially for the prevention of infection. However, a new onset of fever still noted with hypotension, dyspnea, and drowsy consciousness during the hospitalization, prompting surveillance of cultures of urine, deep pus, and blood. Laboratory data revealed an elevated high-sensitivity C-reactive protein (hsCRP) level of 12.08 mg/dL and leukocytosis with a white blood cell count of 13,200/μL, with 62.7 % neutrophils.

**Fig. 2:**
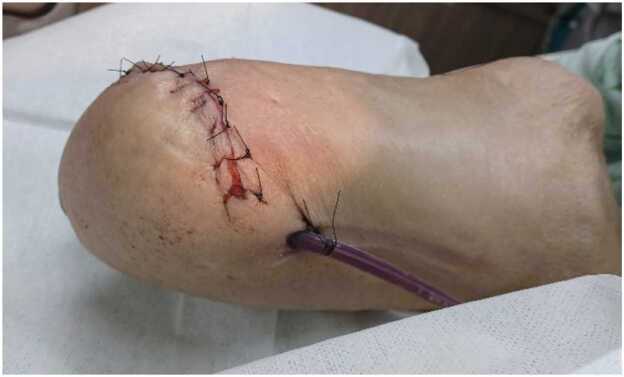
Below-knee amputation was performed to control the infection and progressive wound necrosis.

Both deep pus and blood cultures confirmed *P. stuartii* infection; therefore, the patient was empirically treated with intravenous cefepime at 2 g daily, adjusted for renal function. Due to the patient's bedridden status and infection, empirical intravenous antibiotic was continued for sepsis management. The organism was reported to be sensitive to cefmetazole, cefotaxime, cefepime, ciprofloxacin, levofloxacin, trimethoprim/sulfamethoxazole, piperacillin-tazobactam, ertapenem, carbapenem, and amikacin. Cefepime was maintained for one week, and follow-up blood and wound cultures seven days after treatment initiation showed no bacterial growth. Despite this, the wound still healed poorly, the patient's consciousness remained drowsy, and septic shock persisted, requiring increasing oxygen support. The patient was intubated and transferred to the intensive care unit for close monitoring. Further bedside debridement failed to control the necrosis, and below-knee amputation was ultimately performed (Fig. 2). The patient was weaned off the ventilator and extubated after the condition was stable. The total stay in the intensive care unit was 47 days.

## Discussion

*Providencia stuartii* is an opportunistic organism. Although the incidence rate of *Providencia* bacteremia is low in the general population, it can be increased in patient groups with long-term indwelling urinary catheters, especially in elderly patients who are hospitalized or reside in a nursing care facility [2]. In this case, the patient had several risk factors for infection, including bedridden, having a long-term Foley catheter, advanced age, and type 2 DM. Previous studies have highlighted the risk of bacteremia associated with urinary tract infections. In a 12-year-period study in Ohio hospital, 49 patients were found to have bacteremia with *P. stuartii* infection, where 78 % of the cases are above 70 years, 96 % are from a nursing home, while 92 % have chronic Foley catheters on admission [6]. The urinary tract was definitely proven to be the source of bacteremia in 35 patients (71 %) and was the probable source in another 5 patients (11 %) [6]. While *P. stuartii* is more commonly reported in urinary tract infections, in this case, the bacterium was isolated from deep pus and blood cultures, indicating that wound infections can also lead to severe bacteremia.

*P. stuartii* infections were proved to be more difficult to treat in diabetic patients, most of them being healthcare-associated bacteremia which require improved infection control measures [3]. Early antibiotic treatment is essential to prevent septic shock and potential mortality in such vulnerable patients. In 2015, Wie SH. reported that *Providencia* is susceptible to cefepime, isepamicin, imipenem, piperacillin-tazobactam, and amikacin. Empirical treatment should favor third- or fourth-generation cephalosporins over first-generation cephalosporins and fluoroquinolones [7]. Recently, the high virulence, pathogenicity, invasive properties, strong biofilm forming capability and multidrug resistance pattern indicates this bacterium to be a highly potential threat for infections in burn and immunocompromised patients. The evidence of being resistant to carbapenems indicates an alarming condition as carbapenems are considered to be the last line drug to treat *P. stuartii* [8]. While not every *P. stuartii* infection results in severe bacteremia, early treatment and antibiotic adjustments based on culture and sensitivity reports are critical for patients with multiple risk factors for prolonged infection.

In summary, wound infection leading to severe *P. stuartii* bacteremia is rarely reported. However, in patients with poorly controlled DM and multiple comorbidities, early and targeted treatment is essential. Cefepime is a reasonable empiric antibiotic choice. With the increasing prevalence of multidrug resistance, the severity of *P. stuartii* wound infection should not be underestimated.

## Authors’ contributions

Yen-Ning Chang, Jian-Jr Lee, and Chi Lo contributed to the manuscript conceptualization. Yen-Ning Chang wrote the original draft, while Jian-Jr Lee and Chi Lo each contributed to editing and revision. All authors read and approved the final manuscript.

## CRediT authorship contribution statement

**Chi Lo:** Writing – review & editing. **Jian-Jr Lee:** Writing – review & editing. **Yen-Ning Chang:** Writing – original draft.

## Funding

This research did not receive any specific grant from funding agencies in the public, commercial, or not-for-profit sectors.

## Declaration of Competing Interest

The authors declare that there are no conflicts of interests
